# Early Lessons From Launching an Innovative Community Health Household Model Across 3 Country Contexts

**DOI:** 10.9745/GHSP-D-20-00405

**Published:** 2021-03-15

**Authors:** Daniel Palazuelos, Lassana M. Jabateh, Miry Choi, Ariwame Jimenez, Matthew Hing, Mariano Matias Iberico, Basimenye Nhlema, Emily Wroe

**Affiliations:** a Partners In Health, Boston, MA, USA.; b Division of Global Health Equity, Brigham and Women's Hospital, Boston, MA, USA.; c Harvard Medical School, Boston, MA, USA.; d Partners In Health - Liberia, Monrovia, Liberia.; e Compañeros En Salud - México, Chiapas, Mexico.; f David Geffen School of Medicine at UCLA, Los Angeles, CA, USA.; g Tulane University School of Medicine, New Orleans, LA, USA.; h Partners In Health - Malawi, Lilongwe, Malawi.

## Abstract

Community health workers (CHWs) are integrated into health systems through a variety of designs. Partners In Health (PIH), a nongovernmental organization with more than 30 years of experience in over 10 countries, initially followed a vertical approach by assigning CHWs to individual patients with specific conditions, such as HIV, multidrug resistant-TB, diabetes, and other noncommunicable diseases, to provide one-on-one psychosocial and treatment support. Starting in 2015, PIH-Malawi redesigned their CHW assignments to focus on entire households, thereby offering the opportunity to address a wider variety of conditions in any age group, all with a focus on working toward effective universal health coverage. Inspired by this example, PIH-Liberia and then PIH-Mexico engaged in a robust cross-site dialogue on how to adapt these plans for their unique nongovernmental organization-led CHW programs. We describe the structure of this “household model,” how these structures were changed to adapt to different country contexts, and early impressions on the effects of these adaptations. Overall, the household model is proving to be a feasible and functional method for organizing CHW programs so that they can contribute toward achieving universal health coverage, but there is no “one-size-fits-all” approach. Other countries planning on adopting this model should plan to analyze and adapt as needed.

## Abstract (precis)

Community health worker programs can contribute substantively to health systems working to implement universal health coverage, but there is no one-size-fits-all model. Program leaders should anticipate needing to adapt their plans as local realities demand, but lessons learned in other contexts can provide guidance on how to best proceed.

## Teaser

**Key Findings:**

- Assigning community health workers to households, as opposed to individuals, is a feasible and functional method for structuring how community health can contribute to implementing universal health coverage.

**Key Implications:**

- Program managers should consider how to adapt international best practices for their unique contexts. The Partners In Health experience with implementing a new approach across 3 new program sites in 3 countries suggests that this is best done by both providing clarity on program architecture and also funding mechanisms for frontline program leadership to visit and learn from each other in situ.
- Policy makers should consider how community health programs can be structured as extensions of functioning health systems into households for all diseases and all age groups, as this provides a platform for working effectively toward universal health coverage.

Resumen en español al final del artículo.

## BACKGROUND

Although community health workers (CHWs) are an essential part of the health workforce, how to best incorporate them into health care systems continues to generate considerable debate and innovation. The scope of work given to CHWs often reflects larger assumptions and ambitions; in the era of selective primary health care and vertical funding for only select diseases, CHWs were usually positioned to focus on individual patients, on only a small number of priority conditions, or primarily on prevention efforts. In the recent push for universal health coverage (UHC), CHWs are increasingly called to focus their energies on entire households and individuals with multiple diseases.

### The Household Model at Partners In Health

Partners In Health (PIH), a nongovernmental organization with more than 30 years of experience working in over 10 countries, has responded to the UHC call by incorporating a CHW approach, called the “household model” (HHM), in 3 countries where larger health care system strengthening partnerships already exist. PIH always works within the public sector, and any clinical spaces or materials provided are done in partnership with the national ministries of health (MOHs), with full ownership maintained by the public sector. The HHM approach has been a PIH initiative to demonstrate to MOH colleagues the benefits of providing CHW coverage to every household in a service area to detect, refer, and follow up on a range of priority health issues through regular home visits (Figure). This integrated approach supports the broader needs of entire households and is presented as a feasible and scalable mechanism for further expanding UHC by linking the household to the health system. Although structuring CHW workflow via home visits has historically been a core experience of both successful nongovernmental organization programs (such as the census-based impact-oriented model in Bolivia)^1^ and exemplar national programs (such as in Costa Rica),^2^ PIH set out to adapt and modernize the tools and techniques from such experiences for 3 new contexts. The authors of this article engaged in a robust cross-site dialogue to spark further innovation and improve quality in their respective programs. The most important insights have been captured here via an online collaborative authoring process in which the first author (DP) provided the article's structure, and then authors from each program added text, comments to other authors' additions, and edits. The first and final authors then sculpted a final product that responded to peer reviewers' comments.

**FIGURE:**
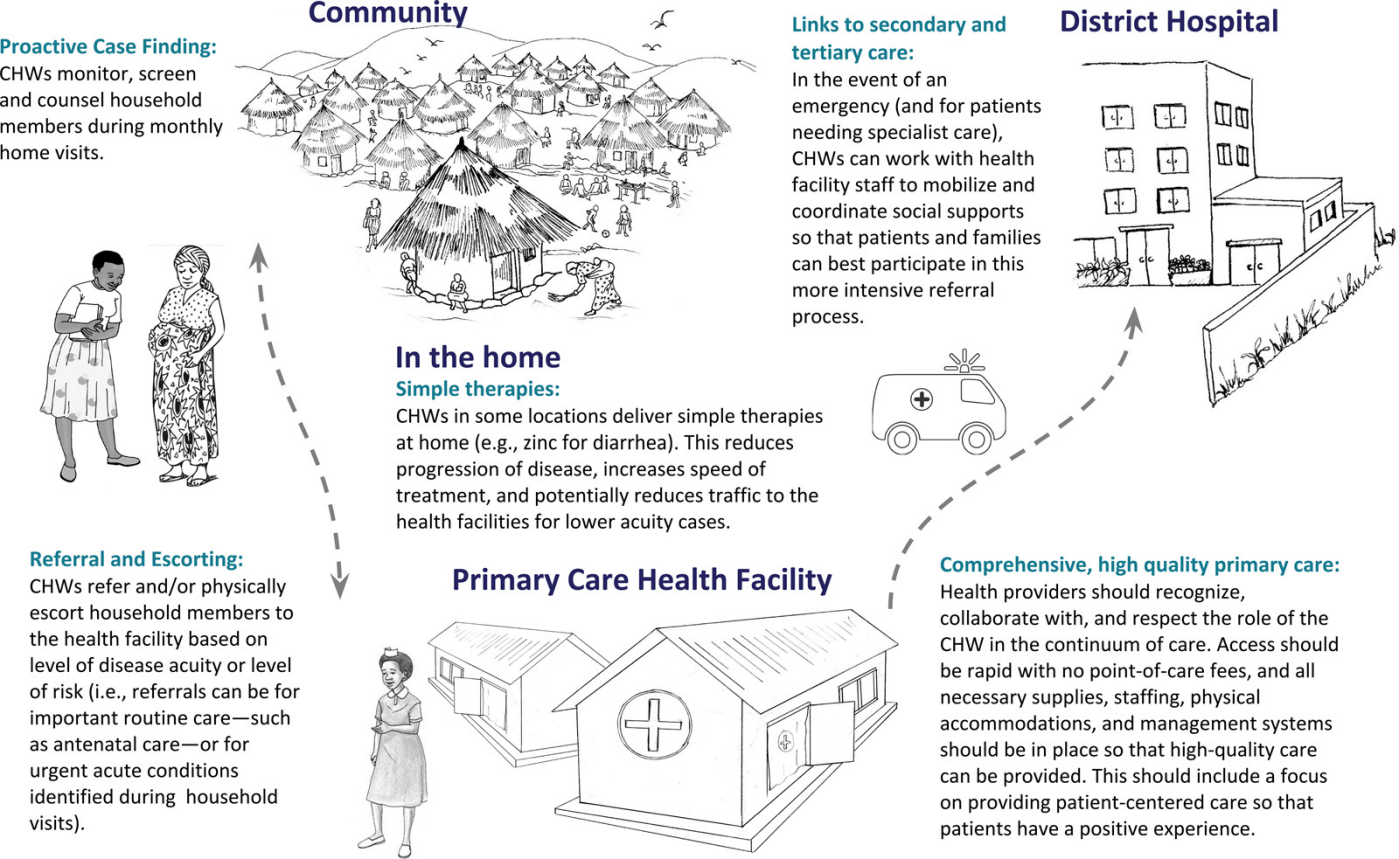
Diagram^a^ of the Partners In Health Household Model Abbreviation: CHW, community health worker.^a^ Cartoon images courtesy of Mango Tree, Jesse Hamm, Petra Rohr-Rouendaal, and Rebecca Ruhlmann.

The household model uses an integrated approach to support the needs of entire households by linking them to the health system–a feasible and scalable mechanism for expanding UHC.

## COUNTRY SPECIFICS

### Malawi

Malawi is one of the poorest nations on the planet, and health spending per capita is only US$35 (as of 2018).^3^ PIH began working in the remote district of Neno, Malawi, in 2007 to support the MOH in responding to the growing burden of HIV and TB. Locally known as Abwenzi Pa Za Umoyo, in partnership with the government, PIH built 2 government hospitals and revitalized several facilities, connecting them to a wide network of CHWs who provided direct support to HIV and TB patients. Combined with efforts in staffing and supply chain, Neno district achieved the highest rate in the country for retention in care for people living with HIV,^4^ improved uptake of maternal services,^5^ greatly expanded care for noncommunicable diseases (NCDs),^6^ and provided services for diseases that are generally undertreated in rural systems, such as Kaposi's Sarcoma.^7^ In 2016, Abwenzi Pa Za Umoyo started transitioning their CHW program to the HHM, in which CHWs were expected to visit each of their assigned households monthly with the goal of becoming the “foot soldiers” for health surveillance assistants (HSAs), the national CHW cadre in Malawi. HSAs largely focus on curative care in health posts, and the HHM supports them by focusing more on active case finding and referral, education, and treatment support for chronic diseases in the home. Data from routine HHM visits are aggregated for monitoring and supervision purposes and reported to the HSAs and the MOH. To determine whether the HHM is effective and to understand any unintended consequences, the team is currently analyzing a stepped-wedge randomized trial of the program.^8^

The HHM in Malawi supports the government-employed HSAs by focusing on case finding, referral, education, and treament support for chronic diseases.

### Liberia

Liberia is also one of the poorest nations on the planet, and health spending per capita is only $US45 (as of 2018).^3^ PIH began working in Liberia in response to the Ebola epidemic of 2014–2016. Committed to supporting the government to rebuild the health system, PIH maintained operations after the epidemic subsided. Similar to Malawi, the PIH team in Liberia revitalized a government rural hospital and connected it to a growing network of CHWs who focused on supporting individual patients with HIV, TB, or leprosy. These CHWs were seen as critical for helping to achieve some of the best clinical results in the country for these diseases.^9^ After the Ebola epidemic, the Liberian MOH launched a community health program that focused primarily on supporting CHWs in remote communities more than 5 kilometers from the nearest health facility. These CHWs— called community health assistants (CHAs)—provide a polyvalent package of primary health care services and epidemic surveillance in 15 counties, serving approximately 29% of the total Liberian population.^10^ The Liberian MOH is currently finalizing a strategic plan to launch another cadre—called community health promoters (CHPs)—for communities located within 5 kilometers of a health facility. Since September 2019, PIH has been a key collaborator in helping to form and refine the strategy for the CHP cadre by partnering with the government to launch a CHP experiment that utilizes the HHM approach to organize how the CHPs engage with beneficiaries within 5 kilometers of 1 PIH-revitalized rural hospital in Maryland county, Liberia. The program's goal is to leverage CHW referrals to increase health facility utilization, improve retention in care, and build trust between the community and the health system. Embedded in the roll-out of the PIH program is a pre- and post-demographic and health survey to provide evidence on the program's impact.

**Figure:**
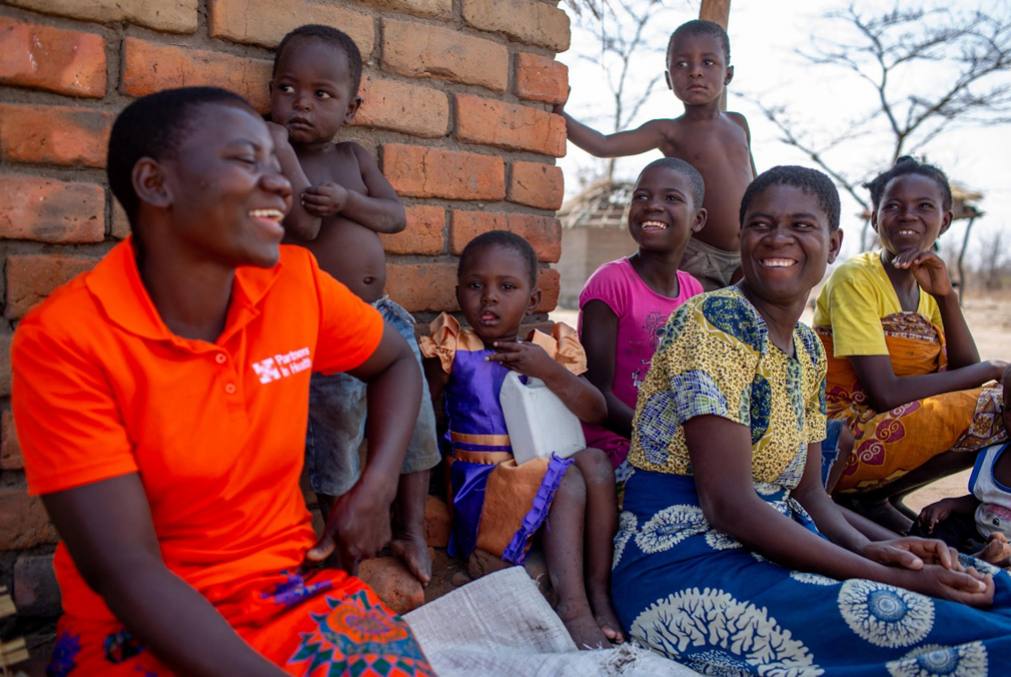
Community health worker Ida Mathala visits the home of Steria Kenoon, 38, and Evance Keneson, 47, in Mtengula Village, Lower Neno, Malawi. The visit covered an noncommunicable disease/diabetes lesson and verbal TB screening. Ida has worked with this family for 3 years and works with 38 other homes in the village. © 2018 Zack DeClerk/Partners In Health

**Figure:**
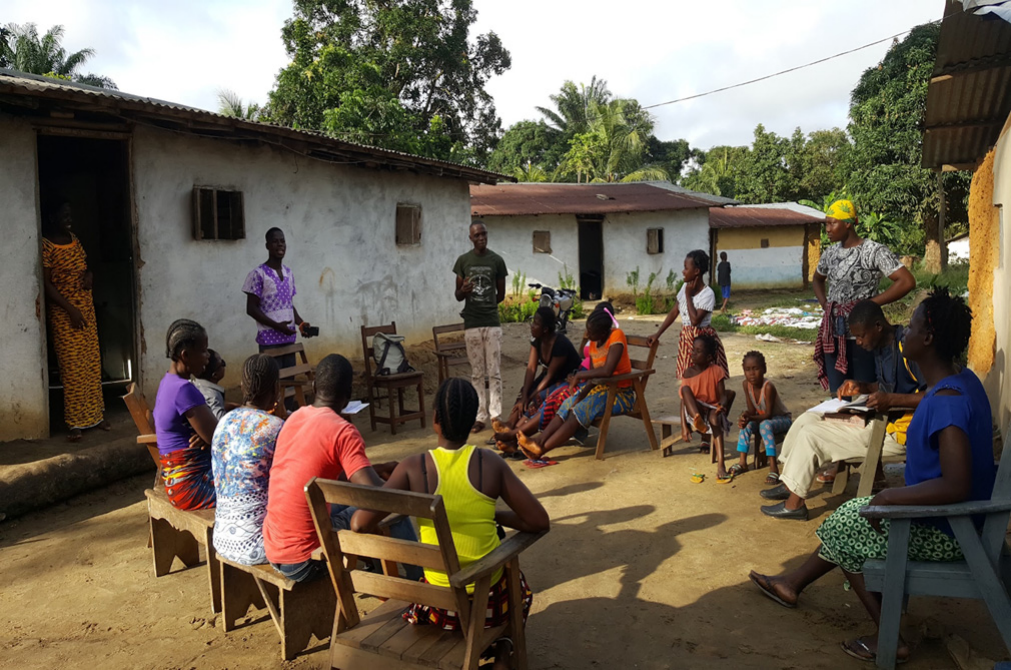
Community health workers Hne-Nma Clark and Jacob Yieh lead a community education session on family planning in Wuduken community, Liberia. © 2020 Miry Choi/Partners In Health

In Liberia, the HHM was deployed to increase health facility use, improve retention in care, and build trust between the community and the health system.

### Mexico

Mexico is an upper middle-income country, but there remain pockets of the country, such as in the southern state of Chiapas, that struggle with extreme poverty and very limited access to quality health care. PIH supported community health efforts in Chiapas, Mexico, for nearly 2 decades before officially launching PIH-Mexico (locally known as Compañeros En Salud, or CES) in 2011. Working with the local health authorities in rural communities in the Sierra Madre mountains, CES revitalized nearly a dozen rural clinics and connected them to 4 cadres of CHWs (locally known as Acompañantes) that were initially focused on providing treatment support for: (1) NCDs, (2) maternal and newborn health, (3) mental health, and (4) child development and nutrition (including early childhood stimulation). Although a stepped-wedge analysis showed that the NCD-focused CHW program had helped achieve some of the best rates of clinical control for NCDs in the region,^11^^,^^12^ the team saw the potential for the NCD and maternal/newborn health cadres to be collapsed into a single cadre through the HHM. In 2019, after regular consultation with the teams in Malawi and Liberia, the Mexico team piloted a new CHW program that moved away from a physician-directed vertical care model, toward a model where CHWs could have more autonomy to use algorithms for screening, follow-up, and referral to the health center.

In Mexico, the HHM helped streamline multiple different CHW cadres into 1 and move toward a model where CHWs had more autonomy.

## THE HHM CROSS-SITE LEARNING INITIATIVE

Teams in Malawi designed the PIH approach on how to assign CHWs to households, including relevant programmatic architecture. They then worked with community health leadership in Liberia and Mexico, both remotely and on-site, to adapt these plans for their contexts. We offer a review and comparison of internal programmatic decisions and iterative adjustments as the programs were adapted to different local realities. Programmatic inputs and parameters decided upon per country are shown in the Table.

**TABLE.** Characteristics of Each Partners In Health Household Model Across 3 Country Contexts

	Malawi: Community Health Workers	Liberia: Community Health Promoters	Mexico: Household Model Acompañantes
Start date	2017	2019	2019
Number Currently Working	1045 CHWs, 183 SCHWs, and 14 site supervisors	46 CHPs, 5 CHP-S, and 1 CHN	70 CHWs (currently being piloted in only 1-2 of 10 target communities)
Catchment Area Served	14 rural catchment areas (138,291 people)	7 peri-urban communities (10,369 people)	10 rural communities (11,645 people)
CHW: Household Ratio	1:20–40	1:40–60	1:30–40
Frequency of Household Visits	Monthly (daily for patients on TB treatment and those on first year of antiretroviral therapy)	Monthly (more often if there are specific patients getting intensive treatment support)	Monthly (monthly, biweekly, or weekly, depending on level of chronic disease control)
Assigned Tasks			
Monthly Home Visits	Provide health education Monitor and screen household members for symptoms related to 8 priority health areas (TB, HIV, STIs, NCDs, family planning, maternal and neonatal health, child health and pediatric malnutrition)	Provide health education Monitor and screen household members for symptoms related to 8 priority health areas (community event-based surveillance, reproductive, maternal, and neonatal health, child health, HIV, TB, leprosy and other neglected tropical diseases, mental health, and NCDs)	Provide health education Monitor and screen household members for symptoms related to priority health areas (hypertension, diabetes, family planning, maternal and neonatal health, and child health) Provide basic treatment at doorstep for diarrhea and acute malnutrition in children Identify vulnerable families eligible for social support
Linkage to Care	Refer or physically escort patient to health provider as needed Follow up on missed appointments	Refer or physically escort patient to health facility as needed Follow up on missed appointments	Refer or physically escort patient to health facility as needed Follow up on missed appointments Assist with scheduling appointments in coordination with health facility staff
Additional Visits for Chronic Conditions	Follow-up visit within 48 hours of referral or after escort to health facility Visit as needed for ongoing treatment adherence and psychosocial support	Follow-up visit within 48 hours of referral or after escort to health facility Visit based on color risk code assigned by facility (daily, 8, 4, or 2 visits per month) for treatment adherence and psychosocial support	Follow-up visit within 48 hours of referral or escort to health facility, 5 days after identification of moderate acute diarrhea, or every 2 weeks for moderate acute malnutrition Visit as needed, in coordination with physician, for ongoing support
Outside the Household	CHWs and SCHWs escort their clients to the facility for medication collection at least once a month and help them navigate the health system. Site supervisors support the integrated chronic care clinic, a mobile outreach clinic that provides care and treatment to all antiretroviral therapy and NCD clients under one roof. CHWs, SCHWs, and site supervisors support HSAs activities (i.e., village clinics at catchment area level as required).	CHPs spend 1 day/month at health facility working with CHP supervisors to help patients navigate the health facility and access services. CHPs work alongside health facility staff during Integrated Outstation Outreach, supporting community-based sputum collection for suspected TB patients and community HIV testing.	CHWs deliver prescribed drugs to home, if needed.
CHW Selection	**Step 1:** Mapping of the catchment area (# of households, # of CHWs required). **Step 2:** Nomination by community leadership and community structures (i.e., chiefs, village health committees, area development committees, village development committees, community-based organizations, and HSAs). **Step 3a:** Literacy test, and pre- and post-foundational training test. Underperformers are provided with support and mentorship to be effective on their job. **Step 3b:** SCHWs undergo an extra 2-day training after the 5-day foundational training.	**Step 1:** Mapping of the catchment area (# of households, # of CHWs required). **Step 2**: Nomination by community health committee using established selection criteria. **Step 3:** Literacy and numeracy test, followed by interview to assess for knowledge, skills, and attributes. **Step 4:** Passing grade (70%) of all training modules and demonstration of a minimum level of core competency, as determined by training facilitators via previously publicized criteria.	**Step 1a:** Mapping of the catchment area (# of households, # of CHWs required). **Step 1b:** Existing vertical CHWs given opportunity to remain in household model after consideration of increased workload. **Step 1c:** Open call for potential CHWs disseminated broadly in target communities. **Step 2:** Literacy and numeracy test followed by interview and subjective ranking of applicants based on interest, availability, and aptitude. **Step 3:** Training for twice as many candidates as positions available. Selection of final CHWs based on daily pre- and post-test evaluations, a final practical evaluation, combined with qualitative assessments from multiple team members (supervisors, trainers, CHW coordinator, etc.). **Step 4:** All remaining candidates that completed the initial training retained as substitute CHWs in case of attrition due to maternity leave, illness, etc.
Payment for Time Worked	Monthly continuous stipend: -CHWs: US$23 -SCHWs: US$31 (full-time minimum wage about US$26)	Monthly stipend: -CHPs: US$50 for ∼20 hours/week -CHP supervisors: US$70 for ∼30 hours/week -CHNs: US$313 for full-time (full-time minimum wage about US$105)	Monthly stipend for CHWs US$100 for ∼20 hours/week (full-time minimum wage about US$170)
Training Schedule	5 days foundational training and quarterly 1-day refresher trainings	2 months foundational training, regular refresher trainings	2 weeks of foundational training (theoretical + practical training and mentorship) with monthly continuing education
Supervision and Mentoring	**1 SCHW assigned to 10 to 15 CHWs** 4 CHW visits per month All CHWs visited per quarter using standardized form for feedback 3 households per CHW visited for spot checks **1 site supervisor assigned to 4–19 SCHWs (depending on number of villages in catchment area)** 4 SCHW visits per month All SCHWs visited per quarter using standardized form for feedback 3 households per SCHW visited for spot checks **Supervision Tools:** Checklists written for supervisors that sit at primary health centers to communicate with health care workers and bridge them to the CHWs on a daily basis An organogram, that everyone understands and helped to write, and with lines of communication mapped out Formal systems for missed visit tracking that different service lines can access (be it NCD, HIV, TB, malnutrition, or patients with cervical biopsy results)	**1 CHP-S assigned to up to 10 CHPs** Visit all CHPs monthly Spend 1 day per week at the health facility to help patients referred by CHPs navigate health facility and access health services Facilitate monthly meetings of CHPs to collect data and discuss challenges **1 CHN assigned up to 10 CHP-S** Visit all CHP-Ss monthly Supervise community-based sputum collection for TB suspects, and community HIV testing Restock monthly supplies (reporting tools, family planning commodities, stationary, etc) Spend up to 40% of the time at the facility for clinical screening, addressing relationships between CHPs and health facility staff, supporting patients in accessing services and with care coordination Facilitate training and refresher trainings Represent the CHP program's successes and challenges at weekly health facility review meetings	**1 CHW supervisor assigned to 10 CHWs** Hold monthly group meetings to discuss challenges, reinforce key competencies, restock CHW supplies, and schedule 1:1 mentoring meetings 1:1 mentoring with supervisor once every 3 months using CHW performance indicators and a standardized open-ended form that encourages supportive supervision Observation-based supervision with CHW during household visits using a standardized form and immediate feedback Household spot checks using standardized form Expected to informally gather feedback from health facility physicians and observe group dynamics

Abbreviations: CHN, community health nurse; CHP, community health promoter; CHP-S, community health promoter supervisor; CHW, community health worker; HSA, health surveillance assistant; SCHW, senior community health worker, NCD, noncommunicable disease.

### Benefits of Adopting the HHM

#### Greater Coverage of Multiple Health Conditions, Integration with Health Facilities, Acceptance, and Social Connectedness

In Malawi, expanding from HIV/TB to several other conditions, initial data demonstrate improved uptake of antenatal care, improved communication across health facilities and CHWs, and greater social connectedness. In Mexico, adding clinical tasks to the scope of work (e.g., blood pressure/diabetes screening) led some community members to express seeing greater credibility in the CHWs' work. In Liberia, vulnerable communities within 5 kilometers of a health facility face significant barriers to care beyond geographic barriers; the CHWs' greater presence in households through regular visits is providing a format by which to build greater familiarity with, and trust in, the health care team.

**Figure:**
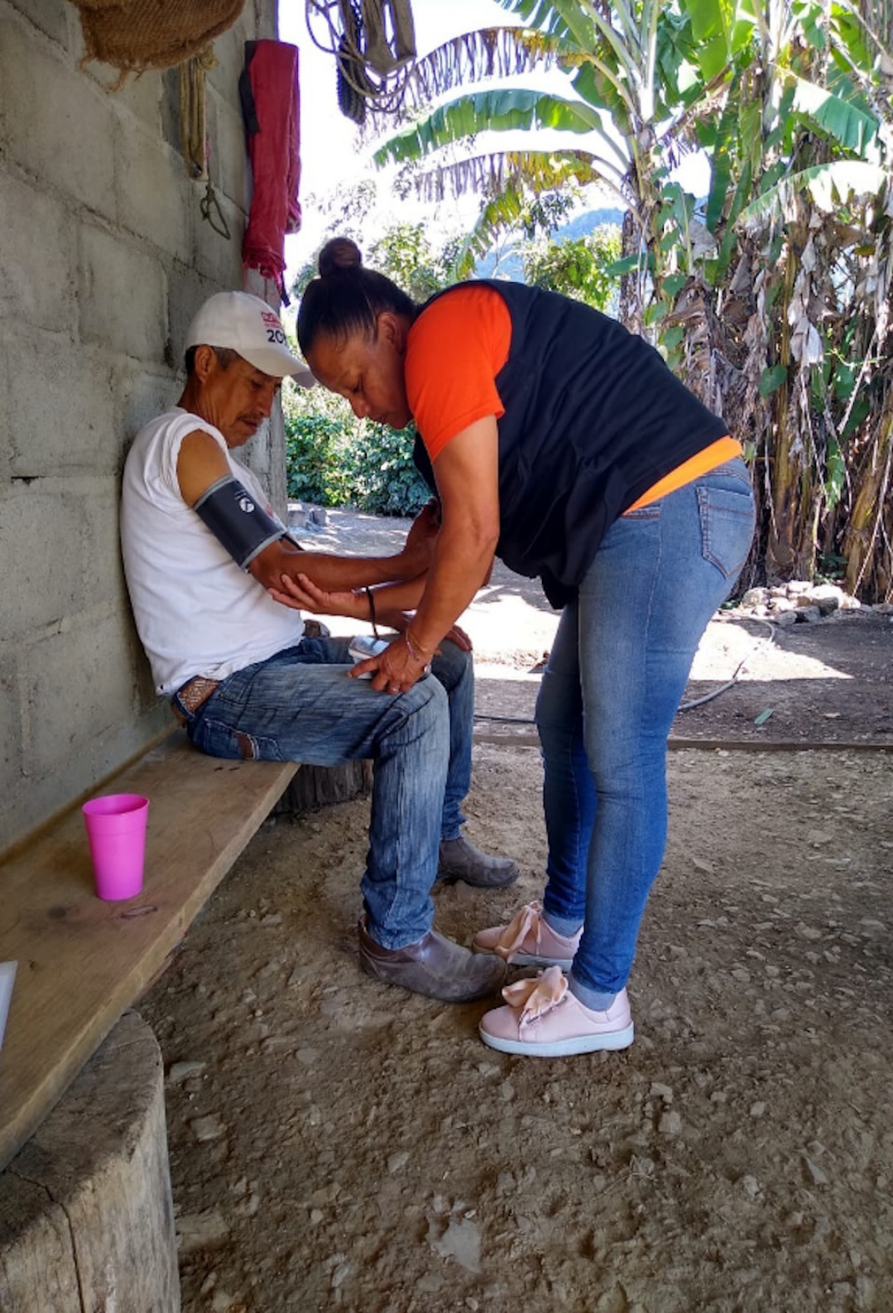
Community health worker Maribel Huerta Luna visits the home of Máximo González López, 61, in Laguna del Cofre, Chiapas, Mexico. The visit covered a hypertension follow-up during the monthly routine household visit. His hypertension is under clinical control, so he receives a visit only once a month. © 2020 Ariwame Jiménez/Partners In Health

#### Greater Opportunities to Focus on “Upstream” Determinants of Health

In Liberia, the arrival of the CHP program presented an opportunity to reengage with previously dormant community health committees in the region where PIH works. These committees were elected by the community to collaborate directly with the CHPs on identifying the community's needs and coordinating health messaging. Embedding CHPs within community social structures increased their legitimacy and opened new opportunities to address public health issues. One CHP, for example, worked with their community health committee to organize a local cleanup of trash.

#### Greater CHW Autonomy

With more disease priorities to focus on, and a wider range of ages, the CHWs have had to seek greater autonomy to address the myriad challenges that arise. In Malawi, for example, the CHWs came to know the complexities of their households such that they could decide which to visit more or less frequently. In Liberia, some CHPs and their supervisors independently planned health outreaches to other communities not yet integrated into the HHM. In Mexico, the CHWs were transitioned from nurse- and doctor-led supervision to a more autonomous unit that coordinates with the local clinic but has freedom to make decisions that affect their workflow.

#### Reduced Stigma for Patients and CHWs

In all 3 countries, a major concern of the patient-centered model was that home visits were a public announcement that someone had a particular disease (e.g., TB or HIV). As such, a number of patients had declined treatment support. In the HHM, a home visit could be for any family member for a wide variety of conditions, so anonymity was inherently easier to maintain. Similarly, because many of the CHWs in the previous model in Malawi and Liberia were themselves living with HIV, a common perception was that all CHWs working with PIH had HIV; this type of bias has not been seen with the HHM.

### Challenges With Adopting the Household Model

#### Imperfect Coordination With Health Facilities Has Consequences

Patient satisfaction with the HHM is often determined by how they are received once referred to the clinic. In Liberia, the CHPs faced difficulties in integrating with the clinical staff at the outpatient department: clinicians would not share patients' diagnoses or treatment regimens with CHPs because of misperceptions that they weren't health providers, and clinicians expressed anger that the CHPs increased the workload with new referrals (but without new investments in the facility). Therefore, CHPs faced both dismissal at the health facilities and in turn frustration from community members who blamed them for negative interactions in the clinic. In Mexico, although CES works actively to improve primary care, similar frustrations were encountered.

#### Encountering Unanticipated Salary Challenges

Moving from a single-disease model to the HHM required increased work expectations, and therefore required an increase in CHW salaries and supervision. In Mexico, however, labor laws were seen by program leadership as a barrier that prevented hiring CHWs full time; paying CHWs a full-time salary as a formal employee added an additional 30% fringe and would have given CHWs the right to be paid a severance fee if the program were to downsize. The PIH-Mexico team was in agreement with the sentiment that CHWs should be afforded such benefits, but current budgeting did not allow for this level of investment. In Liberia, the national community health policy adopted in 2015 that established the CHA cadre also standardized CHA salaries, and as such, PIH had to decrease the CHP cadre salary to align with government regulations. This led to concerns by many CHWs that the salary was not proportional to the increase in work and responsibilities.

#### Transition Pains

In Mexico, CHWs voiced concerns that moving from a single-patient single-disease model to a broader focus with more patients and more conditions diluted and distracted their ability to connect therapeutically. In addition, the new model demanded full-time work year-round, but this was not always possible during the short but intensive coffee harvest period that provided the family with the majority of their yearly income. In Malawi, implementation challenges included long distances to travel between houses, poor transportation options, lack of transportation funds, and demotivation when finding a house empty upon arriving. To address this, households were redistributed to be closer to CHWs' homes. Similarly, in Liberia CHPs received a monthly transportation stipend to assist in physically escorting patients to the health facility, but due to the large demand they could escort only a few before the funds ran out.

#### Managing New Volumes of Data

In Malawi and Liberia, paper data collection systems were aggregated at multiple levels for monitoring and evaluation. This system provided leadership with high level trends but did not allow for real-time analyses to guide decision making (i.e., to adequately support and supervise the CHWs and to measure whether vulnerable populations were reached). As such, Abwenzi Pa Za Umoyo in collaboration with Medic Mobile is piloting the use of mobile health solutions (a smartphone-based mobile application called YendaNafe that enables CHWs to maintain digital records of their work, synchronizing these records in real time with a centralized system). Mexico hopes to learn from this experience to also implement a completely paper-free digital health solution that will include digital elements of supervision.

## LESSONS LEARNED

The HHM is inspired by the principle of “accompaniment,” which is both a philosophical stance and a rubric for programmatic design.^13^ Incorporating community health is guided by 3 principles: (1) CHWs must be professionalized; (2) CHWs must be positioned as bridges to care, not islands; and (3) CHW program budgets must make room for community work and not health work alone.^14^ Although all 3 country programs were philosophically aligned and in regular communication, local adaptations to the HHM were necessary to respond to local realities and pressures. During this process, common lessons arose. Some align with other analyses, such as was described in the CHW Performance Measurement Framework,^15^ and others are unique to this experience.

### Recruit Effectively, With an Eye for Growth

It is important to fully map out what the job entails so the right people can be recruited (i.e., the right attributes, skill sets, geographic distribution, and time expectations). Similarly, if the intervention is to substantially grow in scope, the CHWs recruited need to have the ability to grow with the program through retraining and flexibility with restructuring. This approach is consistent with the CHW development domain of the Performance Measurement Framework. When selecting candidates, the use of written tests and interviews with clear scoring criteria^16^ should be balanced with concerns that literacy criteria might exclude representation from traditionally disenfranchised groups.

### Work to Better Integrate CHWs Into the Health System

CHWs in the HHM are meant to serve as a bridge between facilities and households. Therefore, community members naturally perceive them as an extension of the health facility and sometimes transfer resentment or frustration with the health facility to CHWs. The transition to the HHM has to develop specific and actionable procedures to intentionally engage health facility leadership before, during, and after its implementation.^17^

### Discuss Salary Expectations Early

CHWs should be remunerated fairly through financial compensation.^18^ How much they are paid depends on a variety of contextual factors including local labor laws, other salary benchmarks in the area, and what CHWs perceive as the value of their work. Starting these conversations early and explicitly (i.e., during recruitment), can save considerable debate and discord later. Non-financial incentives may include anything that improves the employment experience (e.g., good training, career growth opportunities, positive interactions with supervisors, etc.) but should be considered and handled separately from salary negotiations.

### Expand CHW Autonomy While Building Functioning Support Systems

To achieve UHC, CHWs will have to be entrusted to operate more autonomously within thoughtfully structured roles in the health system. In line with the CHW Performance Measurement Framework's^15^ goals of job satisfaction, empowerment, and credibility, CHWs should be set up to succeed, including having clear job aids and decision-support algorithms, referral and counter-referral systems, and supportive supervision that empowers them instead of focuses on correcting errors.

### Accept That Trade-offs Are Inevitable

CHW programs hoping to contribute to UHC will inevitably have to make decisions about what is included in the scope of practice and what is not. A common, yet erroneous, tactic seen elsewhere is to simply add more tasks without empowering CHWs to actually take on those tasks, such as by shifting time from other responsibilities or by increasing their salaries so that they can work more hours. If health systems aim to provide coverage for more health conditions, then investments will need to increase to meet new opportunities adequately.

### Cross-national Learning Is Best Done On-site

For organizations hoping to improve multinational sharing and learning, we found that a number of factors facilitated this process. The most useful activity was getting country leadership to visit partner countries. This is in opposition to online conference calls and/or flights to conferences and meetings in the global north. Such on-site “learning trips” build functional relationships, facilitate material sharing, and allow for a deeper understanding of how the programs actually work in their full complexity. What also helped was consistent messaging around how the programs could learn from each other, which reinforced a growing narrative around why the programs were aligned (what has been described by community organizers as “the story of self, us, and now”)^19^ – sites were encouraged to see themselves as connected and then awarded with learning trips when they expressed wanting to connect deeper. This process decentralized the power to teach from a central “expert” in the global north to the network of talented and engaged practitioners on-site.

## CONCLUSION

PIH found that it was possible to adapt the core principles of the HHM across different contexts by utilizing an iterative approach and operational judgment honed from past efforts. The PIH experience with implementing the HHM offers important lessons for other CHW program leaders looking at polyvalent CHW programs embedded within health systems to support progress toward UHC.

## Translation

**En Español**

**Lecciones Iniciales del Lanzamiento de un Modelo Innovador de Salud Comunitaria a Través de Visitas a Hogares en Tres Contextos Nacionales**

**Mensajes clave:** El asignar hogares en vez de individuos a trabajadores comunitarios de salud es un método viable y funcional de estructurar la salud comunitaria para contribuir a la cobertura universal de salud.

**El Resumen**

Los sistemas de salud integran a trabajadores comunitarios de salud (TCS) de manera variada. Partners in Health (PIH), una organización no gubernamental con más de 30 años de experiencia en más de 10 países, inicialmente aplicó un enfoque vertical, asignando TSC a individuos con enfermedades específicas, como VIH, MDR-TB, diabetes y otras enfermedades no transmisibles para brindar apoyo psicosocial y de tratamiento individualizado. A partir de 2015, PIH-Malawi rediseñó sus asignaciones de TCS para enfocarse en todos los habitantes del hogar, cubriendo una variedad más amplia de condiciones en todas las edades, con el propósito de avanzar una cobertura de salud universal y efectiva.

Inspirados por esta experiencia, PIH-Liberia y luego PIH-México iniciaron un firme diálogo internacional con el fin de adaptar el modelo para sus necesidades particulares. Describimos la estructura de este “Modelo Hogar”, los cambios realizados para adaptar el modelo a sus contextos particulares y los hallazgos iniciales producto de estas adaptaciones. En general, el modelo hogar está demostrando ser un método viable y funcional para organizar a los TCS, de manera que avance la meta de cobertura universal de salud. La experiencia indica también que no existe un modelo de “talla única” y que otros países que consideren adoptar este modelo deberán analizarlo y adaptarlo a su realidad.

**Peer Reviewed**
